# Vaccination with Human Papillomavirus Pseudovirus-Encapsidated Plasmids Targeted to Skin Using Microneedles

**DOI:** 10.1371/journal.pone.0120797

**Published:** 2015-03-18

**Authors:** Rhonda C. Kines, Vladimir Zarnitsyn, Teresa R. Johnson, Yuk-Ying S. Pang, Kizzmekia S. Corbett, John D. Nicewonger, Anu Gangopadhyay, Man Chen, Jie Liu, Mark R. Prausnitz, John T. Schiller, Barney S. Graham

**Affiliations:** 1 Laboratory of Cellular Oncology, National Cancer Institute, National Institutes of Health, Bethesda, Maryland, United States of America; 2 School of Chemical & Biomolecular Engineering, Georgia Institute of Technology, Atlanta, Georgia, United States of America; 3 Viral Pathogenesis Laboratory, Vaccine Research Center, National Institute of Allergy and Infectious Diseases, National Institutes of Health, Bethesda, Maryland, United States of America; Imperial College London, UNITED KINGDOM

## Abstract

Human papilloma virus-like particles (HPV VLP) serve as the basis of the current licensed vaccines for HPV. We have previously shown that encapsidation of DNA expressing the model antigen M/M2 from respiratory syncytial virus (RSV) in HPV pseudovirions (PsV) is immunogenic when delivered intravaginally. Because the HPV capsids confer tropism for basal epithelium, they represent attractive carriers for vaccination targeted to the skin using microneedles. In this study we asked: 1) whether HPV16 VLP administered by microneedles could induce protective immune responses to HPV16 and 2) whether HPV16 PsV-encapsidated plasmids delivered by microneedles could elicit immune responses to both HPV and the antigen delivered by the transgene. Mice immunized with HPV16 VLP coated microneedles generated robust neutralizing antibody responses and were protected from HPV16 challenge. Microneedle arrays coated with HPV16-M/M2 or HPV16-F protein (genes of RSV) were then tested and dose-dependent HPV and F-specific antibody responses were detected post-immunization, and M/M2-specific T-cell responses were detected post RSV challenge, respectively. HPV16 PsV-F immunized mice were fully protected from challenge with HPV16 PsV and had reduced RSV viral load in lung and nose upon intranasal RSV challenge. In summary, HPV16 PsV-encapsidated DNA delivered by microneedles induced neutralizing antibody responses against HPV and primed for antibody and T-cell responses to RSV antigens encoded by the encapsidated plasmids. Although the immunogenicity of the DNA component was just above the dose response threshold, the HPV-specific immunity was robust. Taken together, these data suggest microneedle delivery of lyophilized HPV PsV could provide a practical, thermostable combined vaccine approach that could be developed for clinical evaluation.

## Introduction

Despite decades of vaccine development, infectious diseases continue to be the major cause of mortality in much of the world. The roadblocks to global vaccination programs, including those in remote regions, are prohibitive costs of goods and delivery, use of needles, waste disposal, cold-chain requirements, and the crowded vaccination schedule. Solutions to many of these problems involve developing efficient vaccines that are stable at ambient temperatures that can be applied needle-free with minimal waste, and that combine vaccine antigens to reduce the number of inoculations. Here we present a case for a skin-targeted, freeze-dried formulation, combination human papillomavirus (HPV) pseudovirus (PsV) vaccine composed of the L1 and L2 capsid proteins of HPV16 and plasmids expressing respiratory syncytial virus (RSV) antigens.

Delivery to the skin using microneedles poses an attractive means of immunization. The skin is a potent site of immune induction due to the resident Langerhans cells and dermal dendritic cells poised to initiate immune responses to protect the host [1]. Because skin is so rich in antigen presenting cells (APCs), it is an inductive site that may require relatively low antigen doses and a lower requirement for adjuvants. Microneedle patches are an attractive mechanism for delivering biologics to skin, because they can be simply and painlessly applied to the skin for vaccination and other drug delivery purposes. Microneedles are capable of effectively delivering proteins, viruses and nucleic acids in low doses and can be engineered to be stable, strong, dissolvable and highly reproducible [2]. Naked DNA [3–6] and both, influenza [7,8] and HPV [9] virus-like particles (VLP), have been demonstrated to elicit both B and T-cell immune responses after administration to the skin using microneedles. Additionally, using dissolvable microneedles, replication-defective adenoviral vectors [10] and live measles virus vaccine [11] have been successfully administered and induced both CD8^+^ T cell and antibody responses.

HPV, most often HPV16, is the primary etiological agent responsible for cervical cancer [12]. While cervical cancer rates in more developed regions have dropped due to implementation of screening programs, it ranks as the second leading cause of cancer death in less-developed regions where such programs are not available [13]. In addition to cervical cancer, HPV has also been associated with head and neck cancer as well as several other ano-genital cancers [14]. Despite the recent advent of commercial prophylactic HPV L1 VLP-based vaccines targeting the major high-risk cancer-causing types, the high cost of production and requirement for refrigeration make these vaccines inaccessible to most of the individuals in low resource settings, who demonstrate the greatest need for vaccination [15].

RSV is the most frequent cause of lower respiratory tract infections requiring medical care in children under 5 years of age, and there is not yet a prophylactic vaccine available [16,17]. However, there is a monoclonal antibody (palivizumab) specific for the RSV fusion protein (F) that is licensed for prophylaxis in infants at high risk of serious disease [18,19]. RSV F is relatively conserved across subtypes and cross-neutralizing antibodies have been discovered, making it an attractive target antigen for vaccine development [20,21]. As for many other difficult virus targets that do not yet have vaccine solutions, it may be necessary to induce CD8+ T cell responses in addition to neutralizing antibody responses to achieve immunity that can safely prevent infection [22].

In order to elicit complex immune responses, gene-based immunization is appealing because intracellular antigen production results in a protein with authentic structure and processing through both the MHC class I and II presentation pathways leads to a full complement of cellular and humoral immune responses. We have previously reported that HPV PsV, containing both the L1 and L2 capsid proteins, can encapsidate plasmids [23] and deliver genes encoding viral antigens to mucosal tissue successfully eliciting both B- and T-cell responses [24–26]. Precedence exists for microneedle-mediated delivery of plasmid DNA to the skin resulting in expression of the encoded reporter genes and vaccine antigens [4,6]. This, in combination with recent work suggesting that the L1 capsid protein-based HPV VLP vaccine, Gardasil, delivered to the skin with microneedles can elicit neutralizing antibodies (nAb) [9], indicates that HPV PsV may be amenable to this delivery method while retaining their functionality. Here we show HPV PsV delivered to the skin using microneedles can induce immune responses to both the HPV capsid and to the antigens delivered by the encapsidated transgenes in the absence of adjuvant. Use of HPV as a delivery vehicle for plasmids encoding vaccine antigens using microneedles may therefore provide a safe and effective platform for the development of combination vaccines that are easily delivered and may have a much lower requirement for maintenance of a cold chain than currently available HPV vaccines.

## Materials and Methods

### VLP and HPV PsV production

Empty HPV16 L1 and HPV16 L1/L2 VLPs, green fluorescent protein (HPV16-GFP), Luciferase (HPV16-Luc), F protein (HPV16-F) and M/M2 (HPV16-MM2)-expressing HPV16 PsV were prepared as previously described using the 293TT transfection system, purified by density ultracentrifugation using Optiprep (Sigma, St. Louis, MO, USA) and infectious units determined by titrating the preparations on 293TT cells [23,24]. The RSV fusion protein (F) gene was codon-modified based on a consensus sequence of F and was introduced into the same expression vector as M/M2. The sequence can be found here: http://home.ccr.cancer.gov/lco/target.htm.

### Microneedle fabrication, coating and validation

Microneedle arrays, each consisting of a row of five microneedles, were fabricated from stainless steel sheets (Trinity Brand Industries, SS 304, 75 μm thick, Atlanta, GA, USA) by wet etching. The coating solution was composed of 1% (w/v) carboxymethylcellulose sodium salt (low viscosity, USP grade, Carbo-Mer, San Diego, CA, USA), 15% trehalose (w/v) (Sigma, St. Louis, MO, USA) and 0.5% (w/v) Lutrol F-68 NF (BASF, Mt. Olive, NJ, USA) and a vaccine. To increase the coating amount, the HPV-RSV vaccine was concentrated by freeze drying and then reconstituted in coating formulation at the varying concentrations described in each experiment.

The dip coating procedure was performed using a specially designed apparatus, based on a method described previously [27]. The apparatus has a chamber with coating solution and a microneedle holder attached to motorized linear stages (Newmark Systems, Rancho Santa Margarita, CA, USA) allowing the microneedle array to move in two dimensions with micrometer accuracy. The coating was performed automatically while the dipping process was monitored by a video camera attached to a computer. The coating procedure was repeated 8 times to build up a sufficiently thick film. Needles were allowed to dry at room temperature and humidity for 30 s between coating cycles and overnight before use *in vivo*. The amount of coated vaccine was consistent within each batch within 10% accuracy (S1 Fig.).

### Mice and experimental procedures

All animal work was approved by the NIH IACUC. Female 6–8-week-old BALB/c and CB6F1 mice (Jackson Laboratories, Bar Harbor, ME, USA) were housed in the NIAID (VRC) and NCI animal care facilities under pathogen-free conditions and maintained on standard rodent chow and water supplied *ad libitum*, and upon completion of the study, animals were humanely euthanized by carbon dioxide according to the current IACUC-approved documents. BALB/c mice were used for studies assessing HPV immunity and RSV F protein responses, and CB6F1 mice were used for RSV M/M2 studies in order to use the optimal number of tetramer reagents available. Hair was removed from the backs of animals using a chemical depilatory solution (Nair, Church & Dwight Company, Princeton, NJ, USA) 4–6 h prior to microneedle administration. Microneedles were placed for 2 min into the skin on the backs (between shoulders) of anesthetized mice.

For HPV immunogenicity studies, animals received three immunizations, each three weeks apart. Three weeks following the final dose, sera were acquired and animals were cervicovaginally challenged with 10^8^ IU of HPV16-Luciferase (Luc) PsV to determine if they were protected from HPV16 infection [24]. To measure skin expression of luciferase by HPV16-Luc PsV delivery, animals received only a single treatment of either 1, 5, or 10 microneedle arrays (~10^8^ IU/array), bifurcated needles (20 punctures) or needle scratching (10 scratches each from top to bottom and left to right) followed by addition of 10^9^ IU of the same preparation of PsV. Luciferase was detected on days 1 thru 7 post HPV administration by intraperitoneally delivering 100 μl of Luciferase substrate (D-Luciferin Potassium Salt, 15 mg/ml; Perkin Elmer, Waltham, MA, USA), waiting 12 min, then applying 20 μl of the same substrate locally on the skin. After 3 min, images were acquired on an IVIS 100 (Perkin Elmer, Waltham, MA) for 2 min on medium binning. Standardized regions of interest were created around the positive signal and photons were measured using Living Image 3.0 software (Perkin Elmer).

For HPV16-F PsV studies, animals received three immunizations and were cervicovaginally challenged with HPV16-Luc PsV as described above. Four weeks after the HPV challenge (seven weeks after the last microneedle immunization) animals were intranasally challenged with RSV. In the HPV16-M/M2 PsV study, animals were primed with HPV and after four weeks, either challenged with RSV or boosted intramuscularly (IM) with 10^7^ PFU of rAd5-M/M2 [26]. All RSV challenge studies consisted of challenging animals with 10^7^ PFU of RSV A2 (100 μl) intranasally and evaluating protection as previously reported [28]. Bronchoalveolar lavage was performed as previously described [29], except that 1% BSA in PBS was used. Following the bronchoalveolar lavage procedure, nasal wash was performed by inserting the endotracheal tube through the incision in the trachea into the nasopharynx. The nasopharynx was then flushed with 0.2 ml PBS + 1% BSA, collecting the wash fluid from the nostrils.

### Measurement of HPV16-specific neutralizing antibodies

A detailed protocol of the HPV neutralization assay can be found at http://home.ccr.cancer.gov/lco/neutralizationassay.htm and is described in Pastrana et al [30]. Briefly, HPV16 PsV expressing secreted alkaline phosphatase (HPV16-SeAP) were co-incubated with serially diluted sera for 1 h on ice. The samples were then placed onto pre-plated 293TT cells, where they were incubated at 37°C for 72 h, at which time the supernatant was reacted with substrate (Great EscAPe SeAP chemiluminescence kit, Clonetech, Mountain View, CA, USA). Signal was detected on a Polarstar Optima plate reader (BMG, Cary, NC, USA). EC50 (serum titer) was calculated using the average of 6 wells of no-antibody controls to represent 100% SeAP activity. The serum titer was then defined as the reciprocal value of the highest dilution at which the HPV-SeAP PsV activity had been reduced by 50%. The EC50 was calculated and an inhibition curve was generated using GraphPad Prism software v5.0 (GraphPad Software, La Jolla, CA, USA).

### Measurement of F protein-specific antibody response by kinetic ELISA

RSV F protein was purified from virus-infected 293 cells [31], diluted in carbonate buffer (pH 9.6), and coated overnight at 4°C on 96-well flat-bottom ELISA plates (Nunc, Rochester, NY, USA) at a concentration of 80 ng per well. Plates were washed four times with wash buffer (0.02% Tween-20 in PBS) using an automated plate washer (BioTek Instruments, Winooski, VT, USA), and incubated with blocking buffer (2% BSA in PBS) for 1 h at 37°C. In all, 100 μl of 1:10 diluted test sample and positive control were added to each well in triplicate (two coated wells and one uncoated well). Plates were incubated for 1 h at 37°C, washed and incubated for 1 h at 37°C with HRP-conjugated goat anti-mouse IgG1 (1:18,000), HRP-conjugated goat anti-IgG2a (1:8,000) and HRP-conjugated rabbit anti-mouse IgG + IgM (1:20,000) (Jackson ImmunoResearch Laboratories), or HRP-conjugated goat anti-mouse IgA (1:8,000) (Southern Biotech, Birmingham, AL, USA). Plates were washed with wash buffer four times followed by distilled water. A volume of 100 μl of Super AquaBlue ELISA substrate (eBioscience, San Diego, CA, USA) was added to each well and plates were read immediately using a Dynex Technologies microplate reader (Chantilly, VA, USA). The rate of color change in mOD min ^− 1^ was read at a wavelength of 405 nm every 9 s for 5 min, with the plates shaken before each measurement. The mean mOD min ^− 1^ reading of duplicate wells was calculated, and the background mOD min ^− 1^ was subtracted from the corresponding well.

### RSV titer determination

This was performed as previously described in Graham et al, 1988 [28]. Briefly, the left lung was removed under sterile conditions or the entire nose was excised en block, placed in 10% EMEM, quick-frozen in an alcohol-dry ice bath, and stored at −80°C. After quick thaw, tissues were kept chilled while individual samples were ground using a GentleMACS machine (Miltenyi Biotech, Bergisch Gladbach, Germany) on program Lung 02. Clarified tissue supernatant were inoculated onto 80% confluent HEp-2 cell monolayers in triplicate and overlaid with 0.75% methylcellulose in 10% EMEM. After incubation for 4 days at 37°C, the monolayers were fixed with 10% buffered formalin and stained with hematoxylin and eosin. Plaques were counted and expressed as log10 plaque forming units (pfu) per gram of lung tissue or pfu per nose.

### Blood tetramer staining

Whole blood (250 μl) was lysed in 1 ml ACK lysing buffer (Quality Biologicals, Gaithersburg, MD, USA) for 7 min. Samples were washed with 2 ml PBS and centrifuged at 600 x g for 5 min. Lysing and washing was repeated up to three times until the cell pellet was devoid of visible amounts of RBCs. After final removal of the supernatant, samples were stained according to the protocol for the surface and tetramer staining described below.

### Surface and tetramer staining of lymphocytes

Lymphocytes were isolated from right lung with Fico-Lite (Atlanta Biologicals, Atlanta, GA, USA), and tetramer-stained with K^d^M282–90 or I-A^b^M209–223 and antibodies to CD3, CD4 and CD8 and analyzed by nine-color flow cytometry as previously described [24,32] using ViViD (Life Technologies, Grand Island, NY, USA) staining to exclude the dead cells. The gating strategy has been previously described [24]. Flow Jo version 8.7.3 software (Tree Star, Ashland, OR, USA) was used to analyze the data.

### Statistics

Data were analyzed by one-way statistical analysis of variance. Pair-wise comparisons were made using the Holm-Sidak, Dunn’s method, or Student’s *t-*test. All statistical tests were performed using SigmaStat 3.0 for Windows (Systat Software, San Jose, CA, USA) or GraphPad Prism v5.0 (La Jolla, CA, USA). All plots were generated in Excel (Microsoft, Redmond, WA, USA) or GraphPad Prism v5.0.

## Results

### Immunogenicity and efficacy of HPV16 L1 VLPs coated onto microneedles

To test the feasibility of generating protective immunity against HPV infection using microneedles, we compared intramuscular injection to microneedle delivery of HPV16 VLPs. An additional intramuscular control using VLPs rinsed from microneedles by vortexing them in a 1.5ml tube containing sterile PBS was included in order to ensure that the conformation-dependent VLP epitopes that induce neutralizing antibodies were not compromised by the freeze drying and microneedle coating process. HPV16 VLP immunization was performed with a high dose (3 μg) and a low dose (0.3 μg) in the absence of adjuvant. Animals were immunized three times, each three weeks apart. Three weeks following the final immunization, sera were collected to measure anti-HPV16 neutralizing antibody (nAb) titers and animals were then intravaginally challenged with HPV16-Luc PsV as a means for determining efficacy of *in vivo* protection.

**Fig 1:**
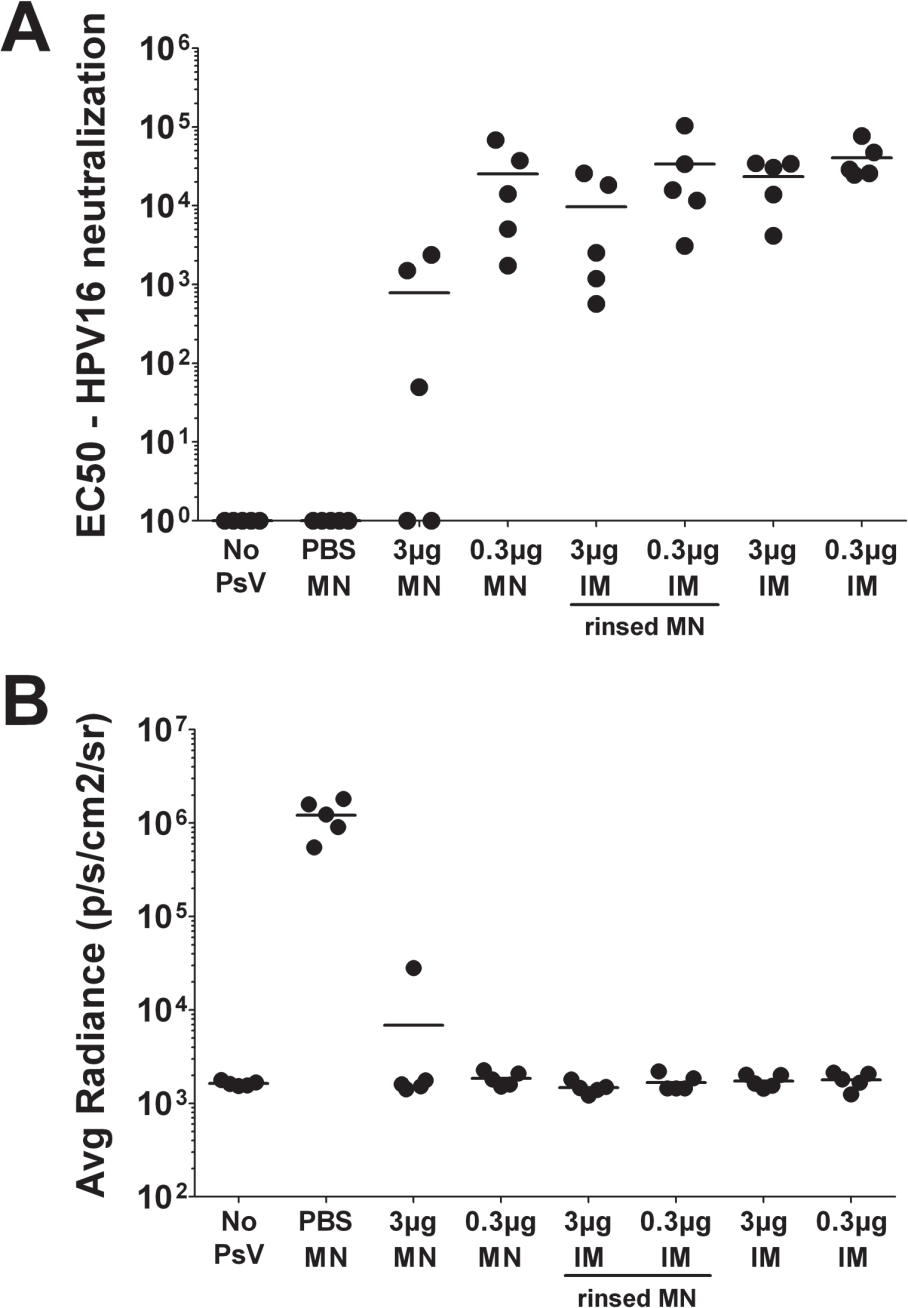
HPV16 L1 VLPs are immunogenic after coating onto microneedles. Animals were vaccinated with two different doses (3μg and 0.3μg) of HPV16 L1 VLPs by microneedle (MN), or intramuscular (IM) injection. An additional group composed of animals receiving an intramuscular injection of VLPs rinsed off of the microneedles was added in order to ensure immunogenicity was retained during the coating process. (A) Neutralizing antibodies against HPV-16 as measured in the sera of animals on the day of cervicovaginal challenge. Data reported as EC50, the reciprocal dilution at which 50% of the pseudovirus was neutralized compared to positive control wells. (B) *In vivo* bioluminescence after challenge with HPV16Luc (HPV PsV encapsidating the firefly luciferase gene). The “No PsV” group was not challenged in order to serve as a negative control group for luminescence. Data reported as average radiance; n = 5.

At the 0.3 μg dose of VLP, vaccination using microneedles or IM needle and syringe injection, either with unprocessed vaccine or vaccine eluted off of the microneedles, generated similar levels of nAb (Fig. 1A). At the 3 μg dose, antibody titers in the IM injection groups were similar and not significantly higher than those in the 0.3 μg dose groups. Animals receiving the 3 μg dose coated onto microneedles displayed lower neutralizing titers compared to the other groups, likely attributed to the difficulty in administering the thickly coated microneedles to the animals. Regardless of vaccination route or dose, the vaccine-induced nAbs were both highly and similarly effective *in vivo* as demonstrated by protection from cervicovaginal challenge with 10^8^ IU of HPV16-Luc PsV (Fig. 1B). Of note, one animal in the 3 μg microneedle group was not completely protected from challenge, and this animal was one of the two with undetectable levels of nAbs.

With the exception of the 3 μg microneedle group, nAb titers were similar among the groups indicating: 1) the immunogenicity of the VLPs remains unaffected by the microneedle coating and application process; 2) there is no dose response signifying that even at 0.3 μg of antigen, the antibody response following microneedle delivery is equivalent to IM immunization with the same or with a one log higher dose; and 3) microneedle delivery is comparable to intramuscular delivery for inducing a nAb response against HPV.

### Presence of L2 and encapsidated DNA do not interfere with HPV immunogenicity when HPV PsVs are applied by microneedles

*In vivo* HPV PsV-mediated gene delivery requires both the HPV major (L1) and minor (L2) proteins [33]. The current HPV vaccine formulation contains L1-only VLPs, therefore it was important to determine whether HPV L1 neutralizing immunogenicity was not adversely affected by the presence of L2 or encapsidated DNA. Microneedles were first coated with a PsV preparation encapsidating a plasmid encoding the RSV fusion protein (HPV16-F PsV) yielding approximately 10^8^ IU of PsV/array. Microneedles were rinsed into PBS and the L1 content was measured by Western blot and determined to be ~25 ng L1/array (S2 Fig.). HPV16L1-only VLPs and empty HPV16L1/L2 VLPs were then coated onto microneedles to achieve a similar concentration per array. Animals were immunized with 10 (250 ng), 3 (75 ng) or 1 (25 ng) microneedle array(s) three times, each three weeks apart. Three weeks following the final immunization, sera were collected and animals were cervicovaginally challenged with HPV16-Luc PsV to measure protection.

**Fig 2:**
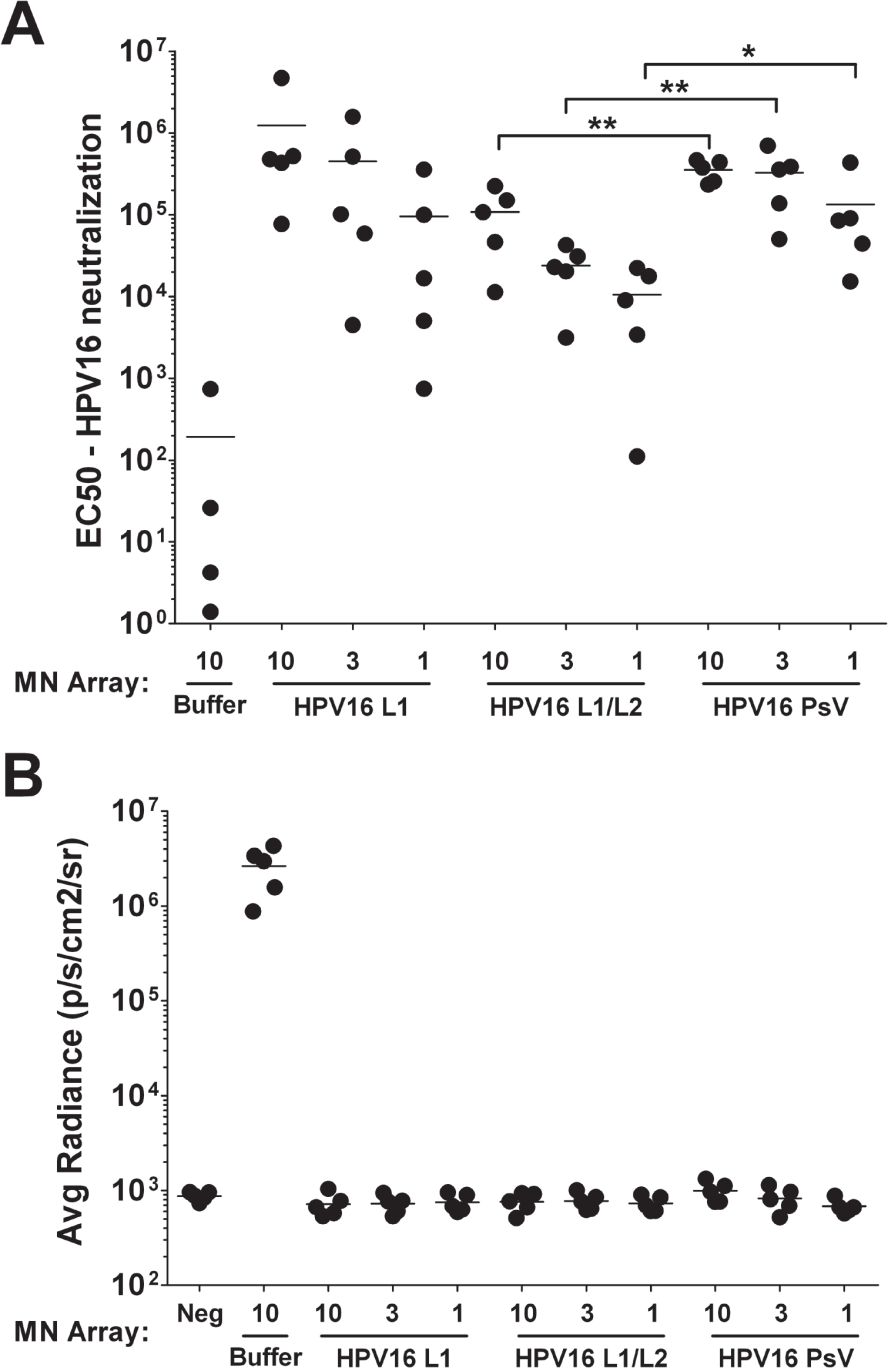
HPV16 mmunogenicity is retained by inclusion of L2 and DNA using the HPV PsV. Animals received one of three variations of the HPV16 particle: L1 only, L1/L2 empty particles or L1/L2 encapsidating the RSV F protein expression plasmid (HPV16-F PsV). Approximately 25 ng of HPV L1 equivalent was coated onto each microneedle array (MN Array), and mice received either 10, 3 or 1 microneedle arrays. The control arm received 10 microneedles coated with coating buffer only. Animals were immunized three times, each three weeks apart. Three weeks after the last immunization, serum was drawn and mice were cervicovaginally challenged with 10^8^ IU of HPV16-Luc. (A) nAb were detected in all groups and a dose response was observed. Data reported as EC50, the reciprocal dilution at which 50% of the pseudovirus was neutralized compared to positive control wells. (B) Protection from challenge was observed in all groups as measured by *in vivo* bioluminescence after challenge with HPV16-Luc. The “No PsV” group was not challenged in order to serve as a negative control group for luminescence. Data reported as average radiance; n = 5. *p = 0.03, **p<0.01.

High levels of nAb were detected in all groups tested, regardless of dose and presence of L2 or DNA (Fig. 2A). A dose response was noted in each of the three conditions examined. A statistically significant drop in nAb levels was observed in the animals receiving empty L1/L2 particles when compared to the PsV immunized animals, however, all mice were completely protected from an intravaginal challenge of 10^8^ IU of HPV16-Luc (Fig. 2B). This difference in anti-L1 antibody elicitation may be attributed to the presence of the DNA in the pseudovirus acting to stabilize the particles, or acting as an adjuvant to overcome the decrease in titer attributed to the presence of L2. Collectively, these data indicated that the induction of HPV neutralizing antibodies is not diminished when the HPV capsid is configured for use as a gene delivery system.

### Optimization of gene delivery using HPV PsV-coated microneedles

**Fig 3:**
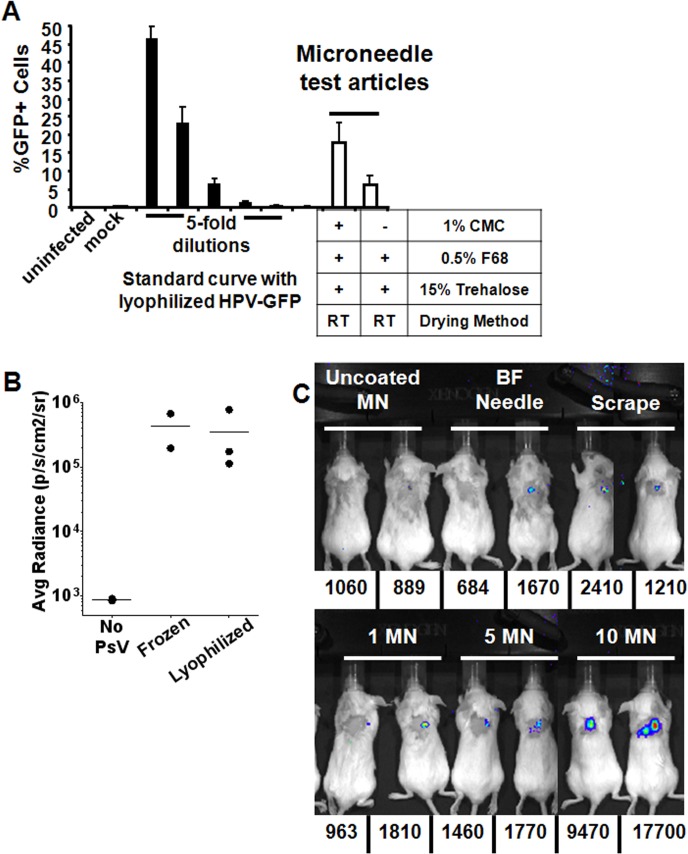
Coating optimization and kinetics of gene expression when delivered by HPV PsV-coated microneedles into the skin. (A) Comparison of frozen and lyophilized HPV16-Luc PsV in the murine cervicovaginal challenge model. Animals were challenged with 10^8^ IU of HPV16-Luc from the same lot that had either been lyophilized or frozen (-80°C). Data are reported as average radiance, n = 2–3. (B) For the microneedle test articles, microneedles were coated with the standard coating formulation as well as a coating formulation lacking CMC (RT = room temperature). HPV16-GFP (green fluorescent protein) was coated onto the microneedles. The coated microneedles were incubated with monolayers of HEp-2 cells, the cells were then examined by flow cytometry 24 hours later to determine the percentage of infected cells. (C) Mice were inoculated with microneedles delivering approximately 1x10^8^ IU (1 MN), 5x10^8^ IU (5 MN) and 1x10^9^ IU (10 MN) of HPV16-Luc PsV encapsidating a firefly luciferase reporter plasmid. Uncoated microneedles (Uncoated MN) containing no PsV were used as a negative control. For animals receiving the bifurcated needle treatment (BF needle) or the needle scratching treatment (scrape), 1x10^9^ IU of the same preparation was added to the skin immediately after skin traumatization. Mice were imaged daily for luciferase signal and the average radiance is shown below each individual animal. Image and values (average radiance) depict signal observed from animals on day 2 at the peak of luciferase expression, n = 2.

We have previously demonstrated that when delivered mucosally, HPV PsV are efficient vectors for gene-based immunization and that 10^8^ IU of pseudovirus will typically deliver approximately 10ng of plasmid DNA [24]. Prior to testing PsV-based genetic immunization by microneedle, we first validated that freeze-drying the particles and the microneedle array coating procedure did not interfere with the ability of PsV to deliver a plasmid to epithelial cells. After two weeks of room temperature storage followed by rehydration in PBS, the lyophilized luciferase-expressing PsV proved equally effective at gene delivery when compared to previously frozen particles using the *in vivo* vaginal challenge model (Fig. 3A) and HPV16-GFP (green fluorescent protein) particles rinsed from microneedles were capable of delivering a plasmid *in vitro* as indicated by transduction of GFP expression into the cell (Fig. 3B). Additionally, efficiency of *in vivo* particle delivery was examined by inserting PsV-coated microneedles into mice for variable lengths of time, then measuring the amount of particles remaining on the array. Only 20% of the particles remained on the microneedles after 30 s of inoculation (data not shown). Longer inoculation time points did not increase the efficiency of delivery.

To evaluate *in vivo* gene delivery to skin, HPV16-Luc PsV-coated microneedle arrays (approximately 10^8^ IU of PsV/array) were used to inoculate animals for 2 min at doses of approximately 1x10^8^ IU, 5x10^8^ IU and 1x10^9^ IU (1, 5 and 10 microneedle arrays, respectively). Uncoated microneedles, bifurcated needle puncture (20x) and needle scraping (10x horizontal and 10x vertical scratches) served as controls. Beginning 24 h after HPV16-Luc PsV administration, and continuing daily for seven days, animals were imaged for a luminescent signal indicative of successful delivery and expression of the luciferase gene. A luminescent signal could be detected over background in all PsV conditions tested and the transient kinetics of luciferase expression using this method is similar to what has been previously observed in the vaginal challenge model [26,33,34]. In animals inoculated with 10 microneedles, the signal peaked two days post-administration at levels that were much higher than background (Fig. 3C). These data indicate that a dose response is detectable and that HPV PsV coated onto microneedles are capable of delivering a gene *in vivo* to the skin.

Blood was collected from these animals one month after HPV administration, followed by cervicovaginal challenge with HPV16-Luc PsV. Low levels of HPV16 L1 Ab were detected in all groups except the uncoated microneedle and scrape groups (data not shown). Further, *in vivo* protection from HPV challenge was observed in all but these same two groups (S3 Fig.). These data signify that even a single administration of HPV16 PsV-coated microneedles was capable of eliciting sterilizing immunity against HPV.

### Induction of RSV F-specific antibodies by HPV PsV-encapsidated DNA delivered by microneedles

**Fig 4:**
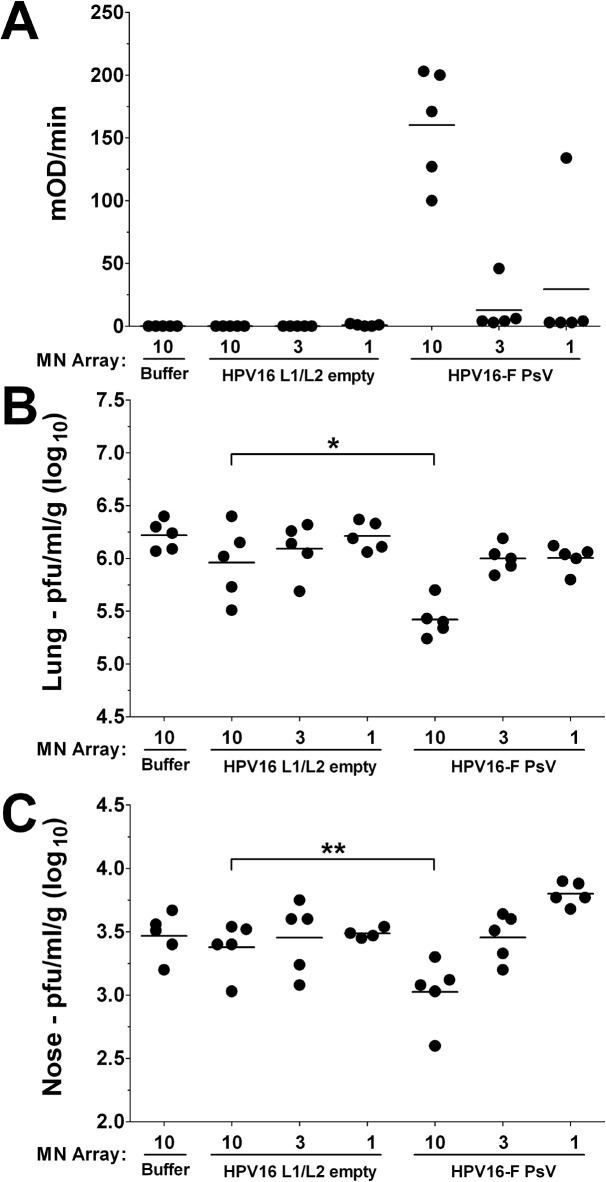
RSV protective antibody responses elicited by microneedle immunization with HPV16- F PsV. (A) Serum antibodies specific for RSV F protein were detected three weeks after the last immunization with microneedle arrays (MN Array; same animals depicted in Fig. 2). Mice were then challenged intranasally with 10^7^ pfu of RSV A2. After 5 days RSV titers were measured in the (B) lung and (C) nose by RSV plaque assays. *p = 0.04, **p = 0.02.

Sera acquired on the day of HPV challenge from the animals immunized with HPV16-F PsV described above were evaluated for RSV F-specific antibodies by kinetic ELISA. Mice receiving the highest dose of 10 microneedle arrays all had F-specific antibodies, whereas, at the lower doses, induction of antibody responses was inconsistent (Fig. 4A). Seven weeks after the last immunization (one month after HPV challenge), animals were challenged with RSV. On day five, animals were euthanized and RSV was quantified in lung and nose (Fig. 4B and 4C). A significant reduction in RSV pfu was observed in both the lungs (p = 0.02) and the nose (p = 0.04) of animals immunized with 10 microneedle arrays coated with HPV16-F PsV.

### Induction of M/M2-specific T-cells by HPV PsV-encapsidated DNA delivered by microneedles

**Fig 5:**
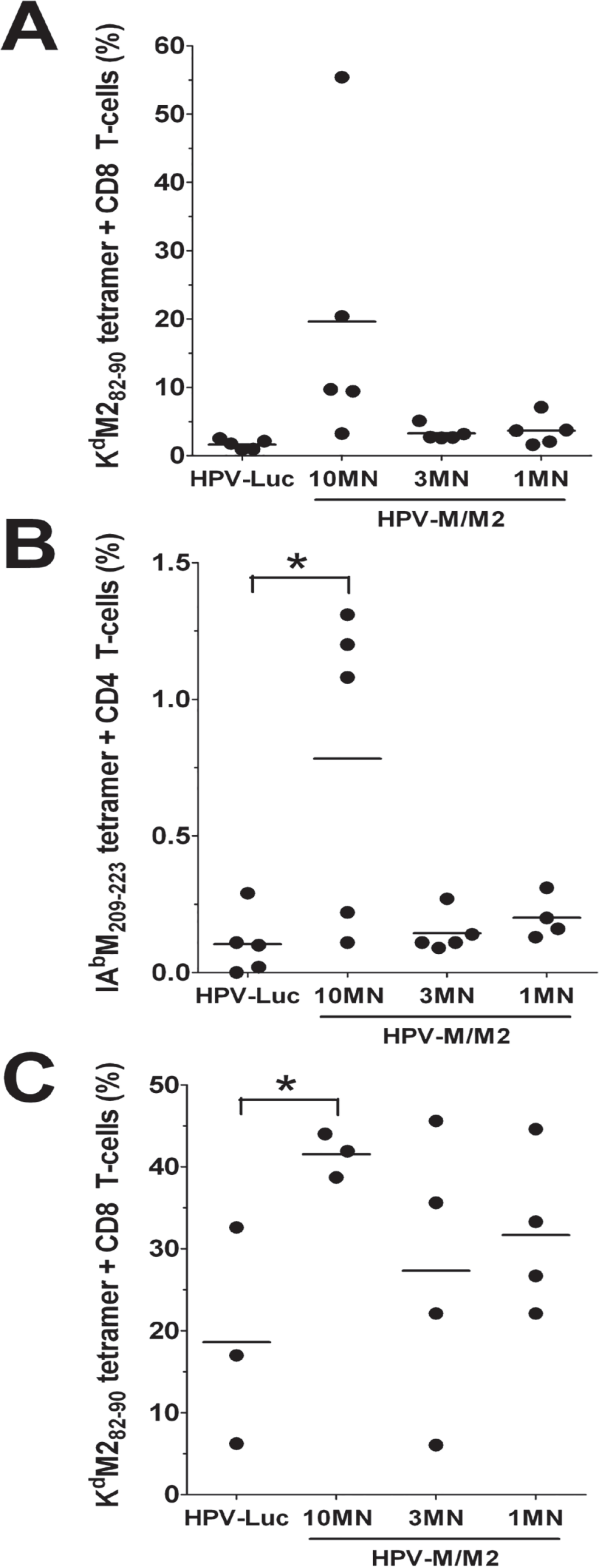
HPV16-M/M2 PsV delivered by microneedles primes for an RSV specific CD4^+^ and CD8^+^ T-cell response. Animals received a single administration of microneedles (10, 3 or 1 arrays) coated with HPV16-M/M2 PsV and the control group received 3 microneedle arrays coated with HPV16-Luc PsV. One month after immunization, mice were challenged with 10^7^ pfu of RSV and five days after challenge, RSV M/M2 tetramer-specific (A) CD8^+^ and (B) CD4^+^ T-cells were measured in the lungs. (C) A second group of animals were boosted IM with 10^7^ pfu of rAd5-M/M2 and CD8^+^ M2 specific T-cells were measured in the blood 14 days later. Data are reported as percentage of tetramer positive cells as determined by flow cytometry, n = 3–5/group. *p = 0.04.

To evaluate T-cell responses induced by HPV PsV-encapsidated plasmids delivered by microneedles, we immunized mice with HPV16-M/M2 PsV, which was previously shown to be immunogenic when delivered intravaginally [24,26]. RSV M/M2 has been used as a model antigen for evaluating both CD4^+^ and CD8^+^ T cell-induced immunity in mice [32,35]. Microneedles were coated with HPV16-M/M2 or HPV16-Luc and groups consisted of 10, 3 or 1 microneedle arrays for HPV16-M/M2 or 3 microneedle arrays for HPV16-Luc. Anti-HPV16 L1 antibodies could be detected by day 25, indicating that indeed the particles were successfully administered (data not shown). As reported for DNA vaccination, antigen-specific T cells are often undetectable after priming. Therefore boosting or viral challenge is required to assess the quality of priming for T cell responses [36–38]. One month after microneedle immunization, animals were divided into two groups: one was challenged with RSV and the other was boosted with a low-dose (10^7^ pfu) of recombinant adenovirus serotype 5 vector (rAd5-M/M2) IM. Epitope-specific CD8^+^ and CD4^+^ T-cells were measured in lungs 5 days post-RSV challenge. Only the groups immunized with 10 microneedle arrays demonstrated higher increases in CD8^+^ T-cell and significant increases in CD4^+^ T-cell responses (p = 0.04) above baseline after challenge (Fig. 5A and 5B). CD8^+^ tetramer responses were measured in blood fourteen days after boosting with rAd5-M/M2 and significantly higher responses (p = 0.04) compared to HPV-Luc immunized controls were only observed in animals immunized with 10 microneedle arrays (Fig. 5C).

## Discussion

The data herein describes the use of HPV PsV as vaccine delivery vehicles for a combination vaccination strategy. The PsV were resistant to freeze drying, lyophilization and coating onto microneedle arrays, retaining both the conformation-dependent epitopes required for inducing neutralizing antibodies and the ability to transduce the genes of other antigens. These findings support the feasibility for using microneedle-based vaccination with HPV VLPs or PsV. Use of microneedles could eliminate the need for typical needle injections and provide a simpler, less painful, safer and potentially more cost-effective approach to vaccination by eliminating the need for cold-chain transportation and storage.

Strong neutralizing immunity was generated against HPV infection after a single dose of approximately 25 ng of HPV protein. Application of the vaccine within the dermal and epidermal layers may be effective at low dose due, in part, to the enrichment of sentinel antigen presenting cells (APCs) that constantly survey the tissue for foreign antigens [1,39]. Langerhans cells and dermal dendritic cells are potent APCs that are directly targeted by the use of microneedle delivery to the skin. These cells represent a continually replenished population of immune stimulators which, once activated, travel to draining lymph nodes to present antigen to T cells. It is not known how the HPV particles were taken up and processed in this study. However, HPV may be especially effective and suited for microneedle delivery as this localizes the viral particles within the epithelium, a context for which they display a natural tropism for basal cells. The stability data and immunogenicity data presented here using PsV combined with the data from Corbett et al [9] suggest that HPV vaccine administration by microneedle arrays should be considered for clinical evaluation.

Immunization with DNA is the simplest form of gene-based vaccination. While delivery and immunogenicity of naked DNA has been improved using devices such as the Biojector [40–42], other approaches including electroporation, are being pursued to improve potency [43–45]. Currently, much of these efforts are focused on IM delivery, and can directly induce or prime for significant T-cell and antibody responses. We have previously demonstrated that, when delivered to the genital mucosa, equivalent immune responses could be elicited with naked DNA compared to a 10,000-fold lower dose of the same DNA encapsidated within HPV PsV [24]. As a non-replicative vector, the HPV PsV is a candidate vaccine that lends itself to highly efficient delivery of nucleic acid to cells in skin.

As opposed to mechanical approaches using ballistic force or electrical disruption to improve transduction efficiency, HPV PsV encapsidation provides a biological solution to facilitate DNA delivery. With targeted microneedle delivery to basal epithelium based on HPV tropism, the current approach has potential to facilitate DNA delivery of genes expressing other vaccine antigens. In this study, we showed that microneedle delivery of HPV PsV to the skin can elicit both antibody and T cell responses against RSV antigens encoded by plasmid DNA encapsidated within the PsV. While the responses were relatively modest, a dose response was observed, and, in animals receiving the highest dosage, an immune response was generated that was capable of reducing viral replication. Co-administering an HPV PsV expressing an immune modifier could potentially enhance the B- and T-cell responses. Expression of the modifier would be transient thus diminishing safety concerns associated with the adverse effects of long-term expression or overexpression of immune modifiers.

To further optimize this vaccination strategy, the primary focus will be on microneedle design and payload in order to deliver a higher dose of DNA. This can most easily be achieved by increasing the number of microneedles on a patch. The microneedle arrays used in this study were small and limited in their ability to deliver higher doses of vaccine compared to more recently developed technology such as patches and hollow microneedles [2]. Also, dissolvable microneedle patches composed of water-soluble excipients represent perhaps the most promising alternative technology and should be examined in future studies [37,46].

## Conclusions

This study addressed three central issues associated with vaccination using microneedles. First, we found that lyophilized HPV VLP or HPV PsV could be applied to microneedles and remain antigenically stable at room temperature. Second, vaccination with HPV VLP generated a robust and protective immune response, supporting the development of microneedle delivery for HPV vaccination in place of IM injection. Third, vaccination with HPV PsV generated strong, protective immune responses against HPV and induced dose-dependent B- and T-cell responses to RSV antigens encoded by the encapsidated DNA. These data support the future development of combination vaccination strategies in which the HPV PsV vector generates immunity against both HPV and the antigen expressed from the encapsidated plasmid DNA. We conclude that vaccination with microneedles may be an ideal method for HPV vaccination as a VLP, and may allow future development of HPV PsV-encapsidated DNA vaccine concepts that would result in immunity to HPV and additional antigens encoded by the DNA.

## Supporting Information

S1 Fig Images of a coated microneedle arrays. (A) A complete microneedle device, including an array of five microneedles at the top and a handle for ease of handling at the bottom. Magnified image of (B) five microneedles and (C) a single coated microneedle under fluorescence optics.
(TIF)

S2 Fig Western blotting for L1 content testing. Amount of HPV L1 protein coated onto each microneedle used in the vaccination experiments was determined by western blot. Ten microneedle arrays and five microneedle arrays from two representative preparations were each rinsed into 50 μl of 1xPBS. Samples were run alongside an HPV16 L1 control standard on a 10% Bis-Tris gel (Invitrogen, Carlsbad, CA, USA). Blots were probed with CAMVIR-1 (1:10,000; Pharmingen, San Diego, CA, USA) and goat anti-mouse-HRP (1:1000; Thermo, Pittsburgh, PA, USA) and developed using Western Lightening Substrate (Perkin Elmer, Waltham, MA, USA). Blot was imaged on an ImageQuant LAS4000 (GE Healthcare, Piscataway, NJ, USA) and quantitated using ImageJ software (http://rsbweb.nih.gov/ij/). Samples are as follows: Lane 1- molecular weight marker (Life Technologies, Grand Island, NY, USA); Lane 2—HPV-16 L1 standard 400 ng; Lane 3- HPV-16 L1 standard 40 ng; Lane 4- HPV-16 L1 standard 4 ng; Lane 5- HPV-16 L1 standard 0.4 ng; Lane 6- HPV-16 L1 standard 0.04 ng; Lane 7–10 μl of 10 MN rinse; Lane 8–5 μl of 10 MN rinse; Lane 9–10 μl of 5 MN rinse; Lane 10–5 μl of 5 MN rinse.
(TIF)

S3 Fig Protection from HPV challenge one month after a single administration of HPV16 PsV. Data showing cervicovaginal challenge of animals described in Fig. 3 one month after administration of PsV by either scraping, disruption with bifurcated needle or microneedles. 10^8^ IU of HPV16-Luc was administered and animals were imaged 48hrs later for luciferase activity. The “No PsV” group was not challenged in order to serve as a negative control group for luminescence. Data reported as average radiance; n = 2.
(TIF)
