# Supplementary figures and images for: Vaccination with Human Papillomavirus Pseudovirus-Encapsidated Plasmids Targeted to Skin Using Microneedles
Source: PLoS One. 2015 Mar 18;10(3):e0120797. doi: 10.1371/journal.pone.0120797 (PMC4364728; doi:10.1371/journal.pone.0120797)

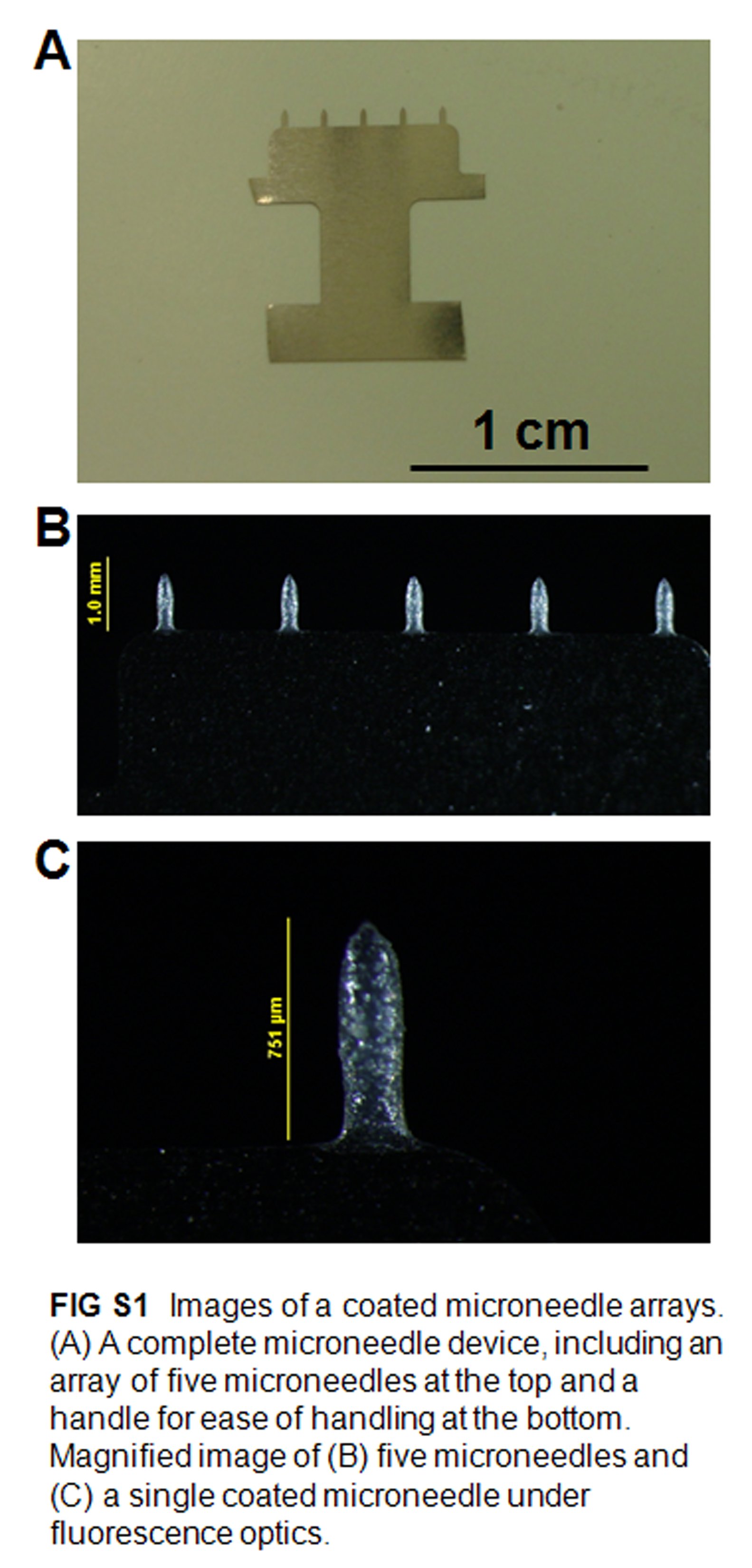

Supplement: S1 Fig — (A) A complete microneedle device, including an array of five microneedles at the top and a handle for ease of handling at the bottom. Magnified image of (B) five microneedles and (C) a single coated microneedle under fluorescence optics. (TIF) [file pone.0120797.s001.tif]

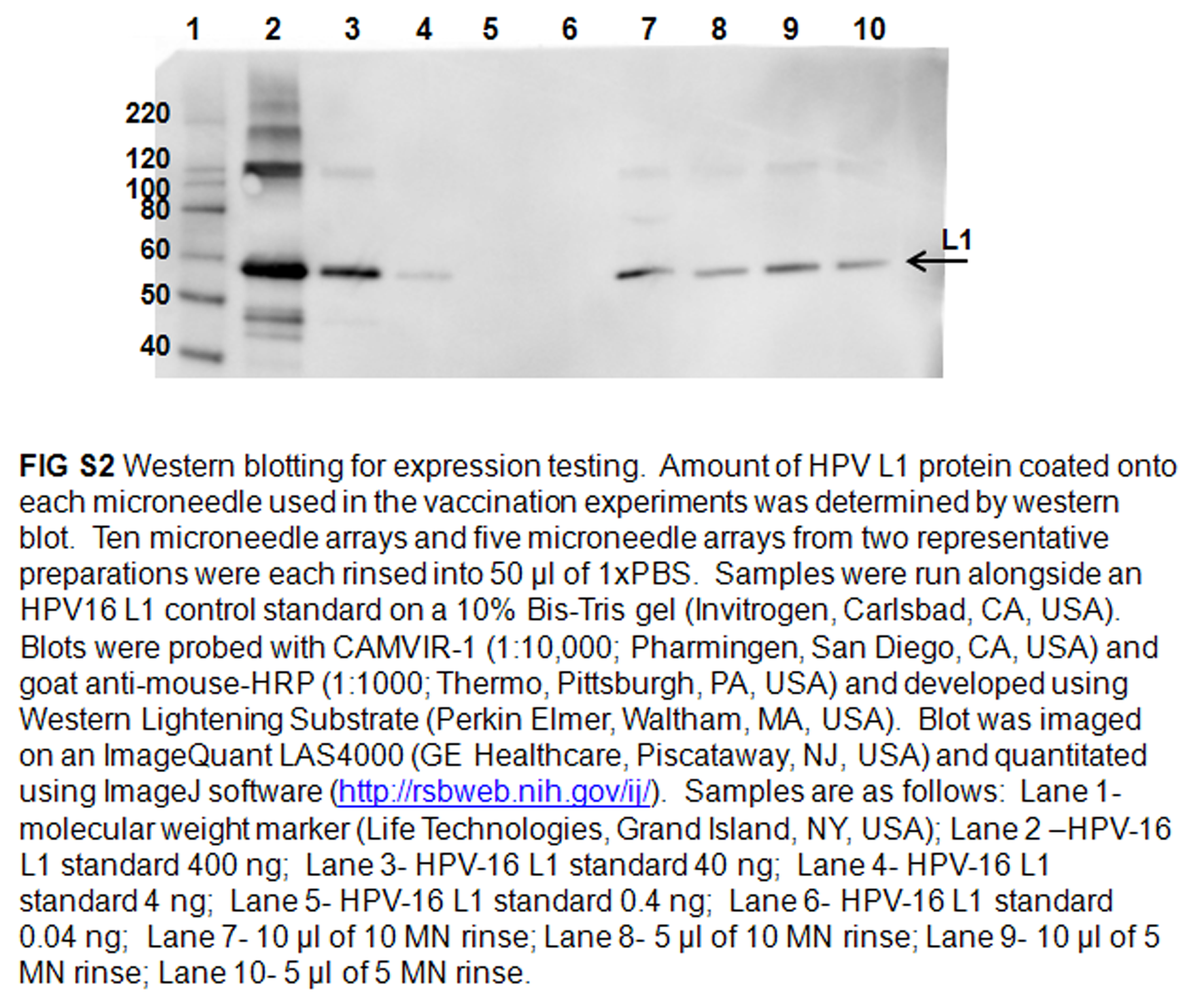

Supplement: S2 Fig — Amount of HPV L1 protein coated onto each microneedle used in the vaccination experiments was determined by western blot. Ten microneedle arrays and five microneedle arrays from two representative preparations were each rinsed into 50 μl of 1xPBS. Samples were run alongside an HPV16 L1 control standard on a 10% Bis-Tris gel (Invitrogen, Carlsbad, CA, USA). Blots were probed with CAMVIR-1 (1:10,000; Pharmingen, San Diego, CA, USA) and goat anti-mouse-HRP (1:1000; Thermo, Pittsburgh, PA, USA) and developed using Western Lightening Substrate (Perkin Elmer, Waltham, MA, USA). Blot was imaged on an ImageQuant LAS4000 (GE Healthcare, Piscataway, NJ, USA) and quantitated using ImageJ software (http://rsbweb.nih.gov/ij/). Samples are as follows: Lane 1- molecular weight marker (Life Technologies, Grand Island, NY, USA); Lane 2—HPV-16 L1 standard 400 ng; Lane 3- HPV-16 L1 standard 40 ng; Lane 4- HPV-16 L1 standard 4 ng; Lane 5- HPV-16 L1 standard 0.4 ng; Lane 6- HPV-16 L1 standard 0.04 ng; Lane 7–10 μl of 10 MN rinse; Lane 8–5 μl of 10 MN rinse; Lane 9–10 μl of 5 MN rinse; Lane 10–5 μl of 5 MN rinse. (TIF) [file pone.0120797.s002.tif]

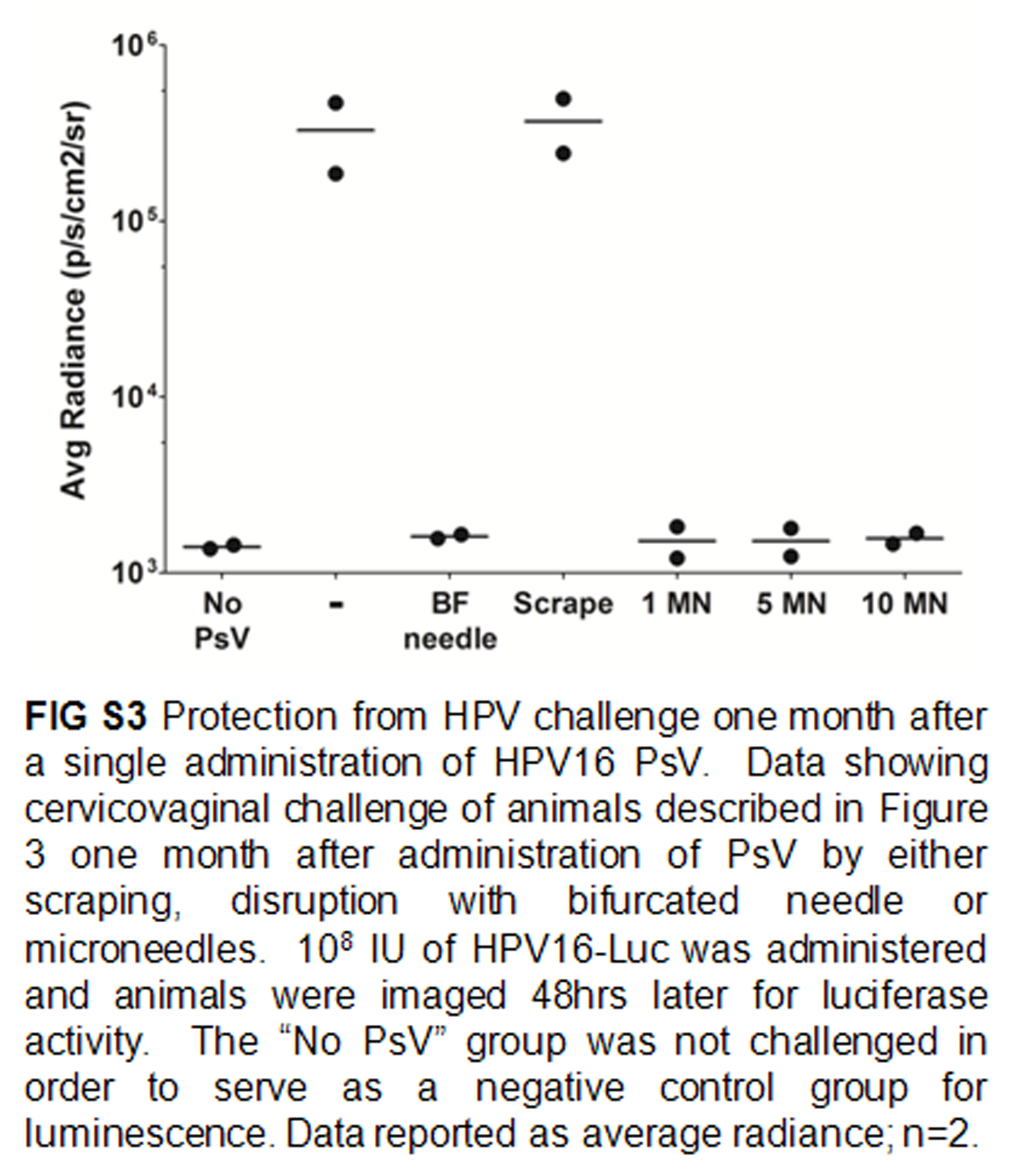

Supplement: S3 Fig — Data showing cervicovaginal challenge of animals described in Fig. 3 one month after administration of PsV by either scraping, disruption with bifurcated needle or microneedles. 108 IU of HPV16-Luc was administered and animals were imaged 48hrs later for luciferase activity. The “No PsV” group was not challenged in order to serve as a negative control group for luminescence. Data reported as average radiance; n = 2. (TIF) [file pone.0120797.s003.tif]
